# Do cool shirts make a difference? The effects of upper body garments on health, fluid balance and performance during exercise in the heat

**DOI:** 10.1186/s13102-023-00768-3

**Published:** 2023-11-14

**Authors:** L. Engeroff, D. Niederer, D. Groneberg, L. Vogt, Tobias Engeroff

**Affiliations:** 1 Department of Radiotherapy and Oncology, Goethe University Frankfurt, Frankfurt am Main, Germany; 2 Institute of Occupational, Social and Environmental Medicine, Goethe University Frankfurt, Frankfurt am Main, Germany; 3 Institute of Sports Sciences, Department of Sports Medicine and Exercise Physiology, Goethe University Frankfurt, Frankfurt am Main, Germany

**Keywords:** Dehydration, Overheating, Heat Stroke, Hyperthermia

## Abstract

**Objectives:**

Due to climate change and major sport events in hot climate, temperature regulation during exercise is gaining relevance in professional and amateur sports. This study compares the effects of an upper body garment with water-soaked inlays, of a synthetic- and of a cotton shirt on health, fluid balance and performance during a high intensity exercise session in the heat.

**Methods:**

32 healthy participants (age 25 ± 4 years; 15 women) were assigned to one of three upper body garments (cotton-shirt, synthetic-fiber-shirt, cooling-vest with water-soaked inlays) and underwent a high intensity steady state ergometer exercise test (Temperature 30.5 °C, frontal airflow 20 km/h, relative air-humidity 43 ± 13%). Time to exhaustion, physiologic parameters (inner ear temperature, heart rate, relative oxygen uptake, body weight, garment weight) and subjective data (perceived exertion, thermal sensation, skin wettedness, clothing humidity, feeling scale) were assessed. Time to exhaustion was analyzed using a survival time analysis. Other outcomes were evaluated using Kruskal-Wallis Tests and 95%-confidence-intervals.

**Results:**

Time to exhaustion was not different between groups. Cooling-vests were heavier and led to lower inner ear temperature, lower thermal- and higher clothing-humidity-sensation at the start of exercise. Physiologic and subjective parameters showed no group differences at exercise termination.

**Conclusions:**

In a realistic setting including frontal airflow, synthetic and cotton-fiber shirts reach comparable effects on health and thermoregulation and are perceived as equally comfortable. Although inducing a small pre-exercise cooling effect, a water-soaked garment induces a weight penalty and creates a less comfortable situation.

## Introduction

Major sport events in the recent past (Olympic Games 2021, Tokyo; FIFA World Cup 2022, Qatar) took place in venues which are challenging athletes with high temperatures and severe air humidity. Due to global warming, increasing heat stress during exercise develops into a global problem for professional and amateur athletes during training and competition.

Heat is a critical factor in sports, because the main part of energy converted by muscular activity is not implemented into movement but thermal energy. Combined with adverse ambient conditions this may cause an escalation of core temperature [1]. The body core does only tolerate a temperature rise of 5 °C [2] before it comes to a severe threat towards vital body functions and a higher risk for mortality [3]. Consequently, high level exercise performance can only be maintained if thermoregulatory mechanisms allow a sufficient level of heat mitigation.

The most effective strategy in hot environments is sweating [3]. Due to fluid loss, plasma volume is reduced and heartrate needs to be increased to maintain cardiac output during prolonged periods with increased sweat rate. These adaptations lead to visceral vasoconstriction, additional load for the cardiovascular system and reduced cardiac output which could limit oxygen delivery to the working muscles and therefore aerobic capacity [4].

Despite the associated health risk, maintaining muscle tissue oxygenation seems to take precedence over temperature regulation in terms of blood flow distribution [5]. During exercise in the heat, this may result in hyperthermia and dehydration; leading to circulatory failure and a spiraling core temperature up to a health threatening or lethal level [2].

Consequently, reducing both, heat stress and fluid loss during exercise is relevant to minimize health risks and enable athletes to perform on an optimal level. Currently applied heat mitigation strategies include heat acclimatization and cooling before exercise as well as fluid ingestion and clothing or technical cooling options during exercise.

One promising strategy for heat mitigation is active cooling throughout exercise (percooling) which offers the opportunity of a larger time frame for cooling and supporting thermoregulating compared to just cooling down athletes prior to competition. Some of these strategies aim at enhanced evaporation and apply fluid soaked inlays in combination with electric fans to increase the airflow during static physical activities [6, 7]. Another approach are pre-cooled vests which showed promising effects on physiologic parameters but require a freezer on-site [8, 9]. Meta-analyses [10–12] already confirmed significant improvements in exercise performance and capacity under percooling conditions. However, no beneficial changes in health-relevant physiologic parameters such as core temperature, skin temperature and heart rate are confirmed by the available literature. Based on the discrepancy between improved performance and unaffected physiological parameters, additional factors such as water loss and sweat production, as physiologic markers for dehydration should be evaluated. Furthermore, the inclusion of heart rate and oxygen consumption offers opportunities to analyze a potential impact of different fabrics on the dissociation of heart rate and oxygen uptake as a result to heat stress [13] and to differentiate effects on cardiovascular strain and aerobic metabolism. Another cause-effect mechanism could be an improvement in subjective perception. Findings on subjective markers are inhomogeneous and currently limited to rating of perceived exertion (RPE) and thermal sensation [10–12]. Additional markers such as skin wettedness, clothing humidity or affective reaction might explain how often advertised effects such as moisture wicking might be related to exercise performance.

Another popular clothing choice is functional clothing made of synthetic fibers such as polyester. Surprisingly, a systematic review concludes that such synthetic fiber shirts show no difference in thermoregulation or comfort when compared to natural fiber clothes [4]. In some studies the synthetic fiber shirt group showed higher core and skin temperature, increased heart rate [14] and a greater fluid deficit [15]. Whereas other experiments report lower end-exercise core and skin temperatures [16] or greater sweating efficiency and less water regain [17, 18] compared to natural fiber shirts. Some methodological limitations could have led to these heterogeneous findings. Temperatures below 30 °C might have put a deficient thermal strain on athletes and thus failed to trigger a sufficient thermal response which would be necessary to evaluate the thermoregulatory impact of shirts or active cooling strategies [4, 11]. Moreover, exercise intensities implemented in several studies are too low to display a realistic setting for intensive training or competition. One factor which has been left largely unconsidered is the influence of airflow on evaporation and subjective perception [19].

Consequently, further studies with challenging but realistic environmental conditions should assess both, the aforementioned health relevant physiological parameters and additional on self-perceived wearing comfort and affective response in order to evaluate further advantages or disadvantages of these established fabrics and newly designed cooling garments. The purpose of this study was to investigate the effect of currently applied synthetic and cotton shirts and an upper body garment with inlays for an increased evaporation on (1) the probability to complete a high intensity exercise session and (2) health related physiological and subjective outcomes in an environment with an increased risk for fatigue, dehydration and cardiorespiratory stress due to heat.

## Methods

This study was conducted as a randomized, controlled, parallel-group experiment. The local ethics committee stated the trial as to be in accordance with the ethical standards set by the Declaration of Helsinki including its modifications [20]. Informed consent was signed by every participant prior to study enrollment.

Adult volunteers were recruited via public tendering in a university setting. All participants were healthy, non-heat-acclimated and reported regular engagement in physical activity (> 150 min per week). Exclusion criteria included functionally restrictive metabolic or acute illnesses. Chronic disease affecting the cardiopulmonary system, infections or drug abuse excluded from participation as well.

A total of 34 participants were included (age = 25; 4 years, height = 1.73; 0.09 m, body weight = 70.3; 13.3 kg).

The trial encompassed a baseline examination at day 1 including documentation of medical history, questionnaire-based recording of participants` health and fitness status as well as anthropometric assessments and a cardio-pulmonary exercise test until volitional exhaustion.

Main examination at day 2 (2–7 days washout in between day 1 and 2) was an endurance exercise test with fixed intensity for a maximum period of 45 min. During both examinations, participants exercised on a bicycle ergometer (Excalibur-Sport, Lode, Groningen, Netherlands). Workload measured in Watt was recorded automatically. Heart rate (HR) was continually measured via chest strap and registered as a 5-second mean value on a corresponding watch (RS800/CX, S810i, S610i, Polar Electro). Respiratory gas parameters were recorded by using a breath-by-breath analyzer (Oxycon Mobile, Viasys Healthcare GmbH, Würzburg, Germany). Again, five second mean values were analyzed. Patients wore a rubber face mask through which the respired air was transferred into a ventilation turbine and further directed to the portable device containing O_2_ and CO_2_ gas analyzers. Relative oxygen consumption (VO_2_) and carbon dioxide output (VCO_2_) data were telemetrically transmitted to a computer. Prior to every testing, the mobile gas analyzing device has been calibrated using reference gases (ambient air, 5% CO_2_, 16% O_2_) as well as automatized standard volume. The breath by breath analyzer was successfully tested for reliability (coefficient of variation for VO_2_ = 3.4, and for VCO_2_ = 4.3) and was compared to the gold standard method to assess validity (Difference of -4.1, 3.1% and − 2.8, 3.5% compared to Douglas Bag method) [21]. According to Perret and Mueller`s recommendation the same spirometry system has been used across all examinations [22]. In addition to that Rate of perceived exertion was assessed in both examinations by using Borg- Scale (RPE; 6 [no exertion] to 20 [maximal exertion]) [23].

Two kind of short-sleeve shirts and a cooling vest have been chosen for the experiment. One of the short-sleeve shirts consisted of 100% cotton whereas the other one was made of 100% polyester with wicking finish (Decathlon, France). Participants were instructed to wear a shirt with a close but comfortable cut and chose the shirt size (ranging from XXS to XL) ad libitum.

The third experimental garment was a sleeveless cooling vest (Idenixx, Germany) providing a tight fit to the torso and integrating front and backside cooling elements. The upper material of the vest was a polyester (83%) elastane (17%) mix and the cooling elements were made of a polyester fleece. Cooling elements were activated by water immersion. Vest evaporization is intended to add to body´s endogenous evaporative cooling.

Volunteers had to undergo a spirometer-based cardio-pulmonary exercise test on a bicycle ergometer to determine individual performance capacity. A ramp-shaped protocol, adjusted to one´s fitness level, was applied to reach volitional exhaustion within 10–12 min. The initial workload was set at 50 W and was increased individually by 10, 15, 20 or 25 W every minute based on participants questionnaire-based report regarding fitness status. The test protocol was in line ACSM´s guidelines for exercise testing and prescription [24]. Participants were allowed to familiarize themselves with the bicycle ergometer and the test protocol.

Criteria defining maximum exhaustion have been: (1) respiratory exchange ratio (RER) > 1.10, (2) achieving age-dependent maximum heart rate, (3) rate of perceived exertion (RPE) via Borg-Scale ≥ 17 [17–20], (4) maximum O_2_ breathing equivalent (< 30) [25].

Maximal oxygen uptake (VO_2_max) was determined by the software by identifying the highest thirty seconds floating mean of oxygen uptake during the whole test [26]. Verification took place manually by the investigator. The parameter was used to ensure homogenous assignment of testing conditions. Participants were ranked according to their VO_2_ max. Groups of three have been formed top down. These groups of three participants were used as stratification grouping for the subsequent block randomization into the three testing conditions.

Respiratory compensation point (RCP) has been detected for each participant by means of the 9 Panels Board and identifying (1) non-linear increase in ventilation (V_E_) compared to linear increasing or non-increasing carbon dioxide emission (VCO_2_); (2) non-linear decreasing end tidal CO_2_ partial pressure (P_ET_CO_2_) as well as an increase in breathing equivalent for CO_2_ [27, 28]. Interpretation of graphic depictions, as described above, is an established approach [27, 28] and has been executed by two independent investigators.

Prior to main examination all participants were instructed to prepare for exercising in the heat via sufficient hydration (minimum of 1.5 L/day; 0.5-liter prior testing). During trial volunteers were not allowed to drink water. After 5-minute-resting phase Bioimpedance Analysis (BIA) was performed by using a tetrapolar device (Nutriguard-MS, Data Input, Darmstadt, Germany) with single frequency (50 kHz). Resistance (R) and reactance (Xc) in Ohms (Ω) were processed by Nutriplus software (Data Input, Darmstadt, Germany). Thereupon body weight was determined by a customary digital scale in kg. Probands have been weighed only wearing underwear and socks. Sport shorts and the randomly assigned upper body clothing option have been weighed separately.

Endurance exercise-test at day 2 was conducted in a room with air conditioning and humidity regulation. We applied standardized hot ambient conditions defined by a temperature of 30.5 °C (tolerable range of 1 °C) and relative air humidity at 43% (tolerable range of 13%). Humidity and temperature were controlled using a thermometer and a hygrometer. During the endurance test the upper body was covered by either one of the three experimental garments. Due to the decisive feel and weight, the testing garment could not be blinded to the participant nor the investigator. Participants performed on the same bicycle ergometer as in baseline examination with identical bike settings as documented within the first examination. They tried to complete a 45-minute ride with the workload of 80% of RCP. Volunteers have been instructed to keep the cadence above 60 rpm. If this limit was permanently fallen below, the test had to be classified as terminated due to volitional exhaustion. Corresponding termination time was noted as outcome (exercise performance in minutes). The time limitation to maximal 45 min of exercise was set due to safety reasons.

In addition to heart rate (beats per minute [bpm]) and oxygen uptake (milliliters per kg bodyweight per minute [ml/kg/min]) inner ear temperature was measured by using a digital infrared ear thermometer (Braun ThermoScan, Mexico) to represent the outcome core temperature (degrees Celsius [°C]). All measurements during all timepoints were conducted by the same investigator using the same thermometer. As self-reported data outcomes we captured Rate of Perceived Exertion via Borg scale (6 [no exertion] to 20 [maximal exertion]) [23] and Feeling Scale (+ 5 [very good] to -5 [very bad]). Moreover sensations regarding temperature (0 [unbearably cold] to 8 [unbearably hot]), sweating (0 [not at all] to 3 [heavily sweating]), clothing humidity (0 [no sensation] to 3 [wet]) and skin wettedness (0 [dry] to 3 [too wet]) [14] were assessed. All outcomes, except Exercise Performance, were documented at rest before the test, at 5 min-intervals while cycling and when terminating the trial. To create a realistic (outdoor-exercise mimicking cycling speed) scenario, airflow was simulated using a fan, located 49 cm in front of the ergometer, directing 20 km/h airflow towards the upper body [29]. Air flow was controlled using a wind sensor.

Statistical analysis was executed by using Prism (Version 9.1.0, GraphPad Software, LLC) and Jamovi (Version 1.6.23.0). A survival time analysis has been implemented by using a 3-group Kaplan-Meier estimator. Log-Rank-test was applied for between groups examination. For both analyses the dependent variable was the duration for individual test termination. Baseline data (cardiopulmonary exercise test, anthropometric measures), pre and post exercise data for objective variables (heart rate, inner ear temperature, VO_2_) as well as self-reported parameters (RPE, Feeling Scale, Thermal-, Sweating-, Clothing Humidity- and Skin Wettedness Sensation) have been analyzed by using Kruskal Wallis Tests (non-parametric analysis of variance due to non-normal distribution of residuals) and Dwass-Steel-Critchlow-Fligner pairwise comparisons (post hoc test). Time series analysis for objective and self-reported outcomes during exercise were conducted on the basis of 95%-confidence interval comparisons for a maximum of nine time points (5, 10, 15, 20, 25, 30, 35, 40 and 45 min) [30]. Pre to post exercise differences in body and clothing weight were analyzed using Student`s t-test. A p-value cutoff of 0.05 was set for significance testing.

## Results

Thirty-two complete sets of data could be enclosed into statistical analysis (6% Dropout). Two volunteers have not been able to pass the main examination due to non-solvable scheduling differences. No adverse events, serious adverse events, or secondary effects occurred. Table 1 displays the sample characteristics and baseline data.

**Table 1:** Anthropometrics, Baseline data and pre and post exercise values of physiologic parameters, body- and garment weight and perceptual data. Data are presented as mean values and standard deviations. VO2 max = maximal oxygen consumption; RCP = respiratory compensation point; bpm = beats per minute; kg = kilogram; cm = centimeter; ml/kg/min = milliliter per kilogram bodyweight per minute

N = 32	Cooling vest		Synthetic fiber shirt		Cotton shirt	
Anthropometric Data						
Sex/gender	male = 6, female = 5, divers = 0		male = 6, female = 5, divers = 0		male = 5, female = 5, divers = 0	
Age [years]	24.3; 2.9		25.1; 4.4		24.7; 4.1	
Body height [cm]	175; 9		172; 9		171; 10	
Bodyweight [kg]	74.2; 13.8		69.0; 14.8		67.4; 10.9	
Bodyfat [%]	25.0; 7.1		25.7; 10.0		26.3; 11.4	
Baseline examination parameters						
Maximal heart rate [bpm]	188; 7		196; 12		190; 10	
RCP heart rate [bpm]	170; 13		173; 14		174; 11	
VO_2_ max [ml/kg/min]	48.6; 7.8		48.3; 11.1		49.4; 8.5	
Oxygen consumption 80% RCP [ml/kg/min]	32.0; 4.2		31.6; 6.6		33.3; 4.8	
Resistance 80% RCP [W]	189; 34		166; 58		185; 60	
Physiologic Parameter prior to exercise (Pre) and at the point of exercise termination (Post)						
	Cooling vest		Synthetic fiber shirt		Cotton shirt	
	Pre	Post	Pre	Post	Pre	Post
Heartrate [bpm]	80; 16	182; 11	90; 11	177; 20	84; 12	182; 13
Oxygen consumption [ml/kg/min]	7.0; 1.9	41.2; 7.0	6.2; 2.2	39.2; 8.7	6.0; 2.4	43.1; 6.6
Core temperature [° Celsius]	36.6; 0.3	37.9; 0.5	37.0; 0.3	37.9; 0.6	36.9; 0.4	38.0; 0.3
Weight prior to exercise (Pre) and at the point of exercise termination (Post)						
Body weight [kg]	73.8; 14.2	73.2; 14.0	68.3; 14.8	67.6; 14.5	66.9; 11.0	66.3; 10.9
Garment weight [g]	842; 291	821; 288	297; 86	358; 154	289; 63	350; 87
Perceptual data prior to exercise (Pre) and at the point of exercise termination (Post)						
Skin Wettedness	0.1; 0.3	2.4; 0.7	0; 0	2.4; 0.5	0.3; 0.5	2.3; 0.8
Clothing Humidity	1.3; 0.6	2.4; 0.9	0.1; 0.3	2.1; 0.9	0.1; 0.3	2.5; 0.7
Sweating Sensation	0.2; 0.4	2.7; 0.5	0.2; 0.4	2.9; 0.3	0.2; 0.4	2.6; 0.7
Thermal Sensation	4.7; 1.9	7.9; 1.1	6.27; 0.6	8.6; 0.5	6.6; 0.5	8.2; 1.1
Feeling Scale	4.2; 1.3	-2.6; 2.0	3.2; 1.0	-1.3; 2.3	3.3; 1.6	-2.6; 2.0
Rate of perceived exertion (RPE)	-	18.8; 1.3	-	17.8; 1.9	-	18.2; 2.1

**Fig. 1:**
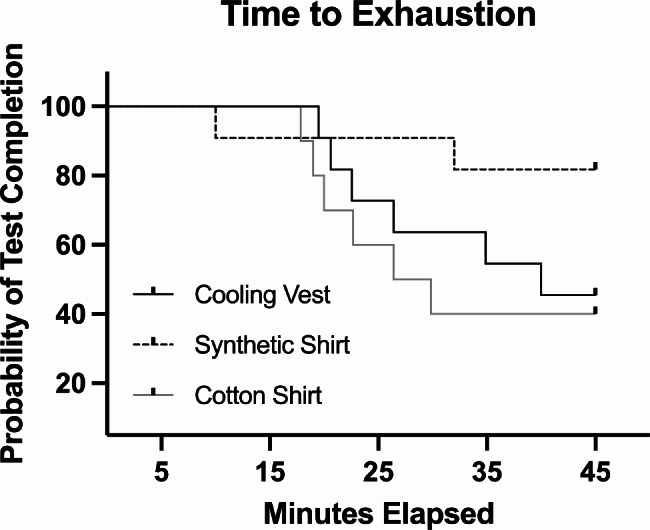
Comparison of the probability of test completion in all three garment groups. Survival time analysis of time to exhaustion in minutes. The horizontal axis shows the time to event and the drops in the survival curve occur whenever a participant stopped exercising

Earliest test terminations occurred in the synthetic fiber shirt group, concurrently within this group most participants managed to complete the desired testing time of 45 min (Fig. 1). The lowest number of premature test terminations was recorded for the synthetic shirt group. Nevertheless, no significant difference in number of premature test terminations between groups had been revealed (Chi^2^ = 4.174, p = 0.124).

**Fig. 2:**
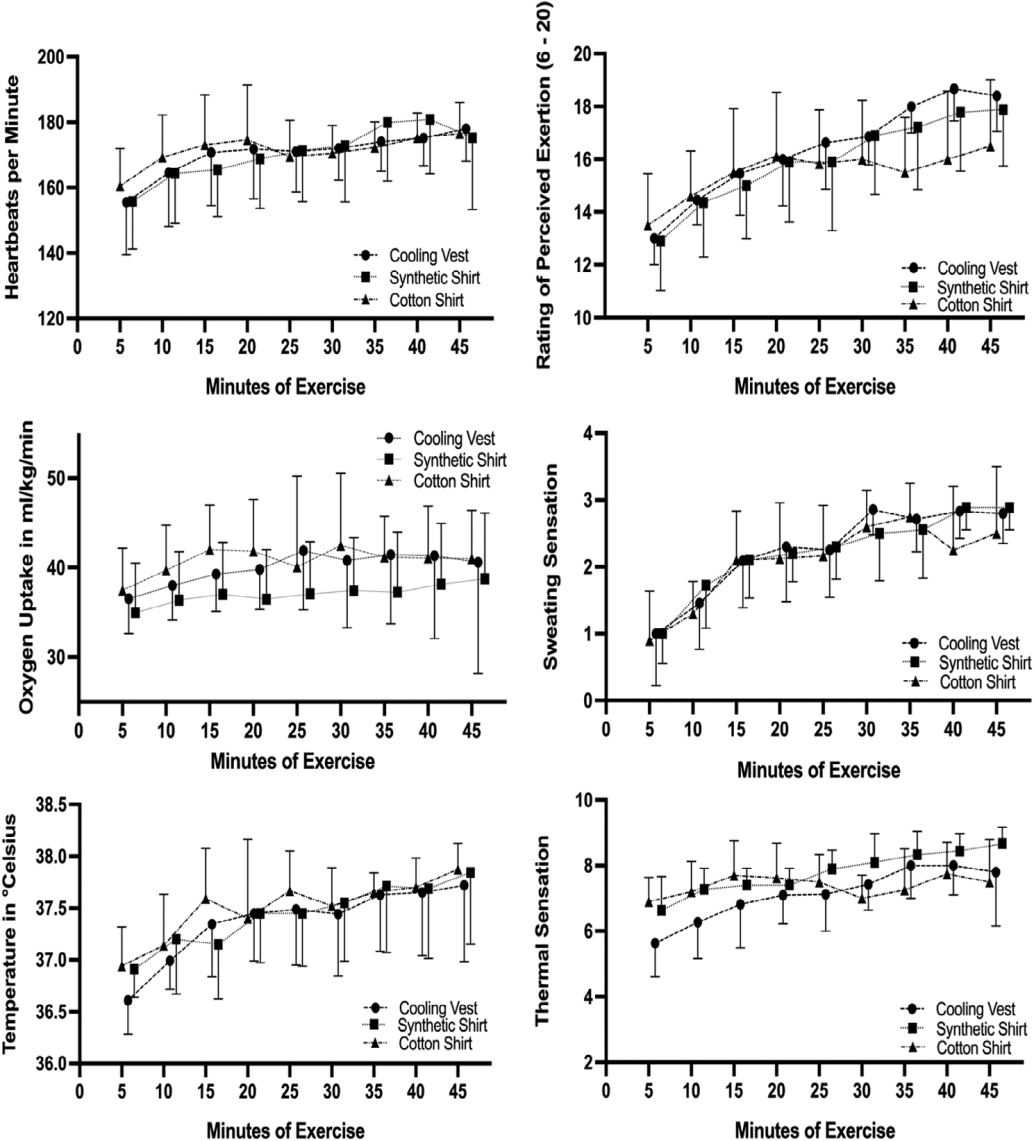
Physiologic outcomes and perceptual parameters during exercise stated as mean values and 95% confidence intervals

Before exercise heart rate (Chi^2^ = 2.539: p = 0.281) and relative oxygen consumption (Chi^2^ = 1.340: p = 0.512) were not significantly different between groups. Core temperature before onset of exercise (already wearing the specific upper body garment) was significantly lower (Chi^2^ = 7.569: p = 0.023) in the cooling vest group compared to the synthetic fiber shirt (W = 3.731: p = 0.023) but not to the cotton shirt group (W = 2.811: p = 0.115). With ongoing exercise-time the heart rate and relative oxygen consumption as well as core temperature increased significantly in each group (Fig. 2). Only core temperature showed significant group differences: After five minutes of exercise, 95% confidence intervals of inner ear temperature within the cooling vest group was significantly lower compared to both shirt groups (Fig. 2). At exercise termination physiologic parameters, including heart rate (Chi^2^ = 0.048: p = 0.976), relative oxygen consumption (Chi^2^ = 0.634: p = 0.728) and core temperature (Chi^2^ = 1.145: p = 0.564) were not significantly different between groups.

Participants` pre and post values regarding body weight and weight of the garment are indicated in Table 1. All participants lost a significant amount of body weight (-0.64; 0.33 kg) from pre to post exercise (T = 11.0; p < 0.05). Body composition analysis indicates that participants with a lower body fat percentage, and thus a higher proportion of muscle mass, lost a greater amount of weight during exercise (rho=-0.493; p < 0.05). However, all three garments showed no effect on differences in bodyweight after exercising (Chi^2^ = 1.78: p = 0.410). The weight of cotton and synthetic fiber shirts was not significantly different before usage (Chi^2^=-0.15: p = 0.99). Although the initial weight of the cooling vests was 350 g, after activation through water immersion the weight of the cooling vest was significantly higher than the weight of cotton and synthetic shirts before (Chi^2^ = 20.65: p < 0.05) and after usage (Chi^2^ = 17.53: p < 0.05)., Exercise showed an effect on the weight variances between garments (Chi^2^ = 7.08; p < 0.05). Whereas the cooling vest lost weight during exercise (-20.3; 123.0 g), the synthetic shirts (28.0; 92.0 g; W = 3.345; p = 0.047) and cotton shirts (35.5; 61.4 g; W = 3.088; p = 0.074) were heavier after exercise. Weight changes in cotton and synthetic fiber shirts did not show a significant difference (W = 0.299; p = 0.976).

Participants` pre and post values regarding perceptual data are indicated in Table 1. Before exercise, thermal sensation (Chi^2^ = 7.268: p = 0.026) was significantly lower in the cooling vest group compared to the cotton shirt group (W = 2.74; p < 0.05) but not compared to the group exercising in a synthetic fiber shirt (W = 2.74; p = 0.128). Furthermore, clothing humidity sensation was significantly higher in the cooling vest group (Chi^2^ = 20.459: p < 0.05) contrasted to the groups wearing cotton- (W=-5.023; p < 0.05) or synthetic fiber (W=-5.223; p < 0.05) shirts. Perception of skin wettedness indicated no significant differences (Chi^2^ = 4.348: p = 0.114) when comparing cooling vests to cotton and synthetic fiber. Sweating sensation (Chi^2^ = 0.0145: p = 0.993) and Feeling Scale (Chi^2^ = 5.194: p = 0.075) showed no between group differences before exercise.

Figure 2 shows 95% confidence intervals for rate of perceived exertion (RPE), sweating and thermal sensation during exercise. All three outcomes present a continuous, significant incline within all three groups. After five minutes thermal sensation was significantly lower for participants wearing the cooling vest than for participants wearing synthetic fiber or cotton shirts. The 95% confidence intervals in Fig. 2 indicate that perceived exertion when exercising in a cooling vest was significantly higher compared to the cotton shirt group after 35 and 40 min of exercise. Considering the point of exercise termination, no significant group differences regarding feeling scale (Chi^2^ = 2.128: p = 0.345), rating of perceived exertion (Chi^2^ = 1.456: p = 0.483), sweating- (Chi^2^ = 1.679: p = 0.432), skin wettedness- (Chi^2^ = 0.020: p = 0.990), clothing humidity- (Chi^2^ = 1.350: p = 0.509) or thermal sensation (Chi^2^ = 2.177: p = 0.337) were detected.

## Discussion

None of the clothing options (cotton shirt, synthetic shirt, cooling vest with water-soaked inlays) allowed a significantly greater proportion of participants to successfully complete a 45-minute training session in high heat conditions. Apart from a significant reduction in inner ear temperature at rest as well as after five- and ten minutes in the cooling vest group, no group differences in physiologic parameters occurred. Fluid loss during activity was comparable in all groups. However, the cooling vest showed a significantly higher weight due to a higher amount of material and fluid storage during exercise. Participants wearing a cooling vest experienced lower thermal sensation before and during the beginning of exercise pointing towards a precooling effect. Nevertheless, a significantly higher clothing humidity sensation pre-exercise as well as greater RPE during exercise in the cooling vest group directs towards a less comfortable situation.

In contrast to current meta-analyses looking at active cooling techniques [11, 12, 31] our findings do not confirm an advantage of a cooling device based on increased evaporation for amateur athletes. Since these earlier studies applied graded exercise tests until volitional exhaustion and assessed maximal workload and physical performance, comparability to our steady state exercise test is limited. Except early time points during exercise, neither the cooling vest nor the synthetic fiber shirt induced beneficial physiological effects. These findings are in accordance with the outcomes summarized in meta-analyses focusing on percooling [11, 12, 31] as well as with a majority of the results which have been presented by Davis et al. [4] in terms of differences between synthetic fiber and cotton shirts. Current evidence regarding fluid loss is sparse and inhomogeneous. Whereas one study documented a greater fluid deficit [15] others speak of a greater sweating efficiency [17, 18] when a synthetic fiber shirt is applied. In terms of active cooling techniques Ruddock et al. [11] and Tyler et al. [12] report few findings which show no effect on exercise induced fluid deficit. Based on our data we confirm comparable fluid loss for cotton and synthetic fiber shirts. Furthermore, a vest with water-soaked inlays showed no effect on fluid loss which indicates that evaporation based on sweat was not beneficially altered. Overall, these findings lead to the assumption that a performance enhancing effect could be induced by subjective factors such as clothing comfort and perceived exertion.

In our experiment self-reported parameters presented significant differences only at the beginning of exercise. At rest and after five minutes participants wearing cooling vests experienced significantly lower thermal sensation. These findings are in line with inner ear temperature data indicating significantly lower temperature of the cooling vest group during the first ten minutes of exercise. We additionally detected a significantly higher sensation of clothing humidity whereas differences in the perception of skin wettedness and feeling scale values did not reach statistical significance. Although thermal sensation is said to be an important mediator of RPE [11], we found no differences in subjective exertion between groups. This might be due to the fact that thermal sensation was altered at the beginning but significant aggravations of RPE occurred during the second half of the test.

Meta-analytic data [11, 12] is limited to thermal sensation and RPE which appeared to have been improved by active cooling strategies. Furthermore, one experiment of Wingo et al. [19] indicated that a synthetic fiber shirt provided significantly better thermal sensation response than the cotton shirt group. In our experiment with simulated airflow cotton shirts seem to have a beneficial effect on perceived exertion during the second half of 45 min steady state exercise. The expanded variety of perceptual variables examined within our study introduces a completely different level of interpretation. We showed that during the period of lower core temperature, volunteers in the cooling vest group experienced a higher level of discomfort generated by a higher sensation of clothing and skin humidity and a decline in feeling scale values. Moreover, at the end of the trial the cooling vest group has been significantly more exerted than the other groups. If we presume that commitment and motivation to perform depend on individual´s sensation of the situation regarding temperature or humidity, perceptual variables may provide the answer to explain the absence of performance improvement. This connection is confirmed by Ruddock et al. [11] who documented the mechanism the other way round, namely that current methods improve performance by benefiting thermal perception and RPE, resulting in greater self-selected external intensities. The negative perception of the cooling vest could be intensified by the fact that the cooling vest was significantly heavier at the beginning and the end of the examination. This may have added to the feeling of uncomfortableness and the unwillingness to perform on a higher level. Additionally, since clothing weight is directly related to performance in weight bearing activities [1], practicability is apparently reduced by this factor, too.

It can be assumed that during the period of lower core temperature and decreased thermal sensation at the start of exercise, volunteers in the cooling vest group experienced a precooling effect based on the water-soaked inlays which lasts for a time period of five minutes. With the vanishing effect, the improvement in thermal perception also disappeared. This leads to the assumption that the cooling vest is a supportive device against external heat stress coming from the environment as long as internal heat augmentation generated by muscular activity and the sweat rate are low. This assumption is in line with the observations made by Tyler et al. [12] as well as Bongers et al. [31] regarding studies examining the effects of cooling prior to exercise. Unfortunately, this effect seems to be of short duration and unable to influence relevant markers of exercise performance.

Although we applied a combination of temperature and humidity which is considered as a potential health hazard leading to fatigue, heat cramps and dehydration according to the heat index of the National Oceanic and Atmospheric Administration, the absence of significant effects in our study compared to earlier experiments could be awarded to a deficit in thermal strain [12, 31]. In contrast to our approach which aimed at simulating exercise in realistic outdoor conditions, authors such as Kenny et al. [8] exposed their participants to uncompensable heat stress using nuclear, chemical as well as biological protective clothing while walking. Furthermore, participants in other studies got to wear ice vests which have been immersed in water and cooled to temperatures between − 24 °C and − 80 °C [8–10]. They documented a remarkable decrease in core (and skin) temperature and an extensive benefit in exercise performance and capacity. Kenny and colleagues` [8] experiment indicates that the magnitude of both, thermal strain and cooling are decisive factors. Bongers et al. [31] also concluded that more aggressive cooling techniques which are employing materials with a temperature below zero have a larger potential to be effective in terms of lowering core temperature and enhancing exercise performance. The cooling vest implemented within our examination incorporated cooling elements which are activated by water immersion (with a temperature between 12 and 15 °C). Nevertheless, concerning the manufacturer water temperature shall not be decisive in terms of the vest´s cooling effect but the supplementary moisture stored in cooling elements. It shall evaporate and therefore add to endogenous evaporative cooling. The differences in terms of applied cooling type and intensity are complemented by the divergent body parts used for cooling. Tyler et al. [12] included studies employing neck cooling during exercise. Bongers et al. [31] and Ruddock et al. [11] reported about studies using head, neck and palm cooling via cool packs. The ingestion of ice slurries, cold fluids and ice slurry mouthwash have been taken into consideration for meta-analysis as well [11]. None of the studies implemented a cooling technique comparable to ours.

Our study aims at analyzing the influence of upper body garments in a realistic setting and therefore applied airflow matched to the cycling speed which would be theoretically achieved during ergometer testing. Our participants did present only a slight increase in internal body temperature during the test and at termination (highest individual = 39.1 °C, highest mean = 38 °C). This implies that thermal stress represented by core body temperature was moderate in our investigation [32]. Authors of meta-analyses on percooling did not mention any trials applying airflow. Anyhow, Davis et al. [4] mentioned a high impact of wind on evaporative and convective heat loss. This has been confirmed by Adams et al. stating a significant reduction in core and skin temperature when raising wind speed from 0.2 ^m^/_s_ to 3 ^m^/_s_ [33]. Wingo et al. [19] used a fan for airflow simulation as well and implemented a speed of 2.4 ^m^ /_s_. They have only been able to detect a significant effect on rectal temperature at exercise termination. Within our examination wind speed was matched to cycling performance and therefore twice as high, at around 5.5 ^m^ /_s_. This supports the assumption of the wind´s decisive influence on promoting cooling via evaporation and convection for all participants. Furthermore, it underlines the need to include realistic wind speeds in future studies on the effect of percooling techniques.

## Conclusion

Since participants exercised on the same metabolic rate (80% RCP) as well as under the same environmental conditions and lost a comparable amount of weight, we conclude that the applied clothing options own similar heat dissipation or cooling characteristics. We thus demonstrated that in a mainly realistic (and challenging) scenario, encompassing high temperature, moderate air humidity as well as natural airflow, neither synthetic fiber nor cotton shirts offer a decisive advantage for non-professional athletes to execute cycling exercise. Furthermore, we were able to show that additional evaporation inlays on the torso do not increase heat dissipation or decrease fluid loss via sweating.

It remains to be further investigated if endogenous thermal stress produced by exercise or external stress formed by temperature and air humidity have not been sufficient to reveal differences between clothing options. Although we did not control the effect of airflow, the comparison with other studies underlines the need to further investigate the potential impact of airflow on heat dissipation and the interaction with cooling strategies. Moreover, it has to be controlled in future studies as a supporting factor which supports convective and evaporative heat loss.

## Data Availability

The data can be requested by the corresponding author Tobias Engeroff. Corresponding email address: engeroff@sport.uni-frankfurt.de.
